# Sintilimab Combined With Albumin‐Bound Paclitaxel and SOX Regimen as Conversion Therapy for Unresectable Advanced Gastric Cancer Achieving Complete Remission: A Case Report and Literature Review

**DOI:** 10.1002/ccr3.73457

**Published:** 2026-09-02

**Authors:** Yufan Tang, Boquan Zhou, Bingbing Wen, Ke Yu, Jiajia Jia, Ying Sha, Jixiang Liu, Luyao Li, Ruifang Fan

**Affiliations:** ^1^ Department of General Surgery The 940th Hospital, Joint Logistics Support Force of Chinese People's Liberation Army Lanzhou China

**Keywords:** albumin‐bound, conversion therapy, gastric cancer, sintilimab

## Abstract

For carefully selected patients with unresectable advanced gastric cancer, immunochemotherapy combining PD‐1 inhibitor with albumin‐bound paclitaxel and S‐1/oxaliplatin may achieve deep remission and enable successful R0 resection. Our findings suggest that this multimodal conversion approach could be considered in selected cases with favorable performance status and MDT‐supported indications, as pathological complete remission appears to be attainable in this context.

## 1 Introduction

Gastric cancer (GC) remains a leading cause of cancer death worldwide, particularly in East Asia [1]. Most patients present with locally advanced or metastatic disease deemed unresectable due to extensive infiltration or distant spread, with overall survival (OS) typically < 1 year [2, 3]. Conventional fluoropyrimidine/platinum chemotherapy has reached an efficacy plateau [4].

The advent of PD‐1 inhibitors has revolutionized advanced GC treatment. Phase III trials (ORIENT‐16, CheckMate‐649, and KEYNOTE‐859) demonstrated that adding a PD‐1 inhibitor to chemotherapy significantly improves OS and progression‐free survival (PFS) versus chemotherapy alone, establishing this combination as standard first‐line therapy [5, 6, 7]. This success has prompted investigation of immunochemotherapy as conversion therapy for initially unresectable GC, aiming to downstage tumors and enable R0 resection, potentially achieving long‐term survival or cure [8, 9].

However, optimal conversion regimens remain undefined. Key challenges include selecting the most effective drug combination, treatment duration, and managing additive toxicities [10]. The combination of sintilimab (anti‐PD‐1), albumin‐bound paclitaxel (nab‐paclitaxel), and SOX (S‐1 + oxaliplatin)—termed “Sintilimab + SAP‐SOX”—has shown promising conversion rates and R0 resection rates in Phase II studies like CO‐STAR, leveraging synergistic immune activation, anti‐angiogenesis, and cytotoxic effects [11, 12]. Nevertheless, its intensity requires careful multidisciplinary team (MDT) oversight due to risks of hematological toxicity and liver injury.

Herein, we report a case of initially unresectable mucinous papillary adenocarcinoma of the gastric antrum with lymph node metastasis that achieved pathological complete remission (pCR) and R0 resection after Sintilimab + SAP‐SOX conversion therapy, with sustained postoperative survival. This case provides real‐world evidence for PD‐1 inhibitor‐based conversion therapy and underscores the critical role of MDT management.

## 2 Case History/Examination

A 55‐year‐old man presented in July 2023 with a self‐palpated upper abdominal mass for 3 months, progressively enlarging with postprandial discomfort. Abdominal ultrasound suggested a gastrointestinal origin, prompting admission.

His medical history included coronary heart disease with acute anterior myocardial infarction and hypertension 5 years ago, treated with percutaneous coronary intervention and long‐term nifedipine, aspirin, and atorvastatin. No family history of cancer.

Physical examination revealed a firm, slightly tender 10 × 8 cm mass in the upper abdomen. Laboratory tests showed markedly elevated carcinoembryonic antigen (CEA) at 1136.78 ng/mL (normal < 5.00 ng/mL); other tumor markers were normal.

**FIGURE 1:**
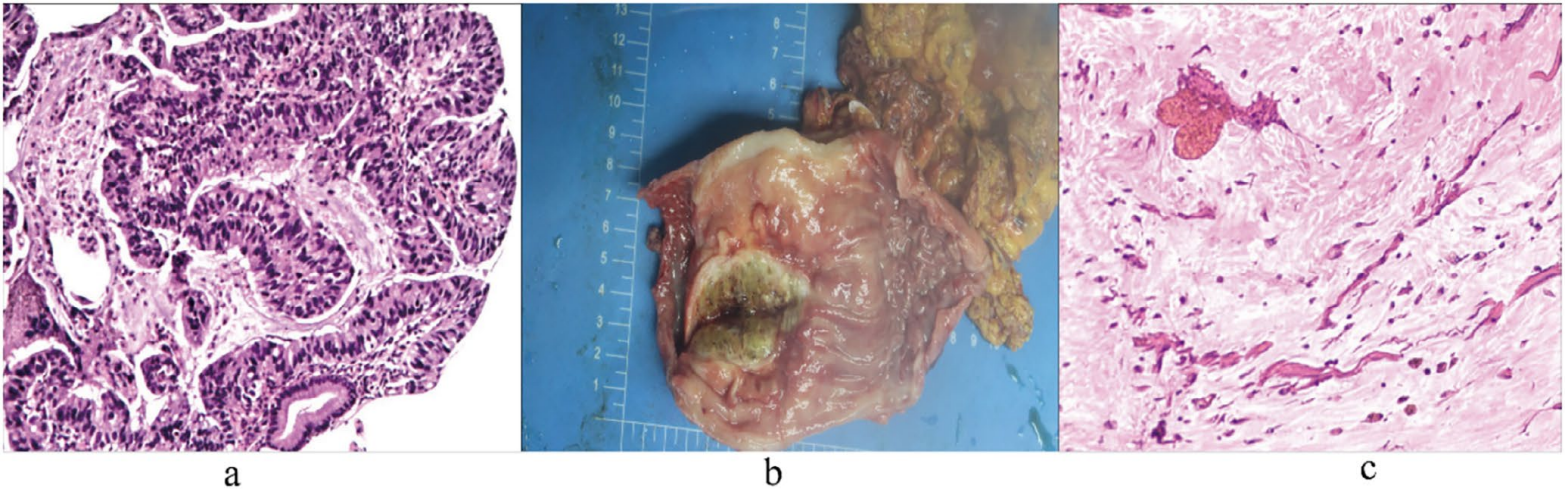
Hematoxylin–eosin staining and immunohistochemical evaluation of the obtained using a pathological biopsy. a: The preoperative pathology image showed mucinous papillary adenocarcinoma of gastric antrum; b: The specimen of the entire stomach, lymph node and tumor tissue removed during surgery; c: The postoperative pathology image showed no residual tumor cells.

Pathological biopsy (August 23, 2023) confirmed mucinous papillary adenocarcinoma of the gastric antrum (Figure 1a). Immunohistochemistry: CKp (+), CD34 vessels (+), Ki67≈60%, CD4 (+)≈20%, CD8 (+)≈20%, HER2 status was assessed and confirmed negative, PD‐L1 CPS≈5%, MSS (MLH1+, MSH2+, MSH6+, PMS2+).

**FIGURE 2:**
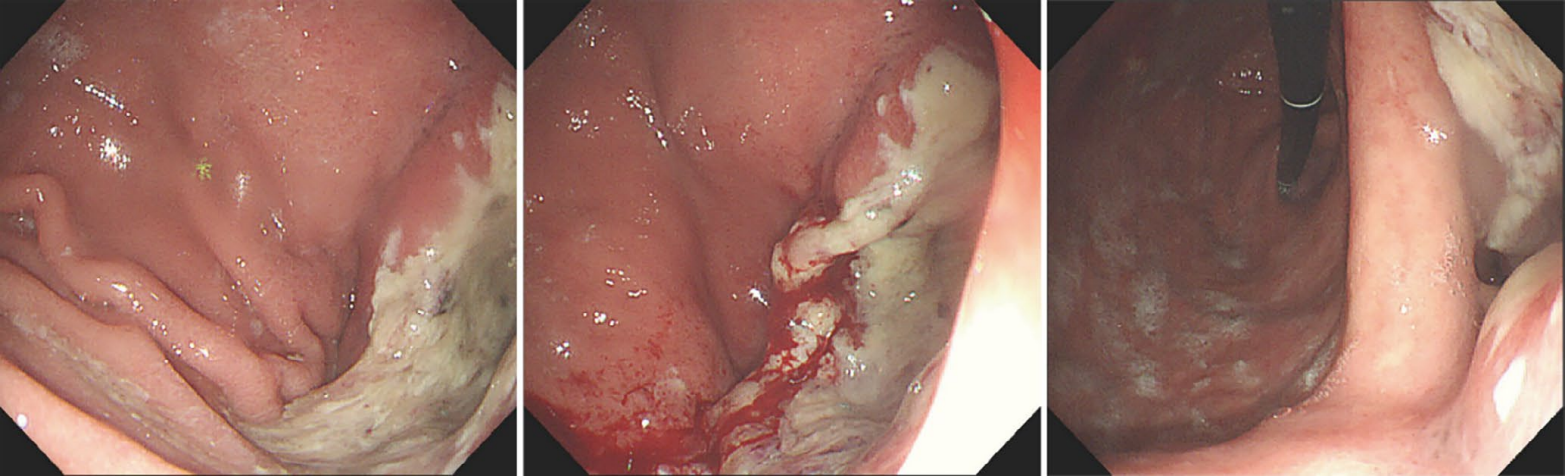
Endoscopic images.

**FIGURE 3:**
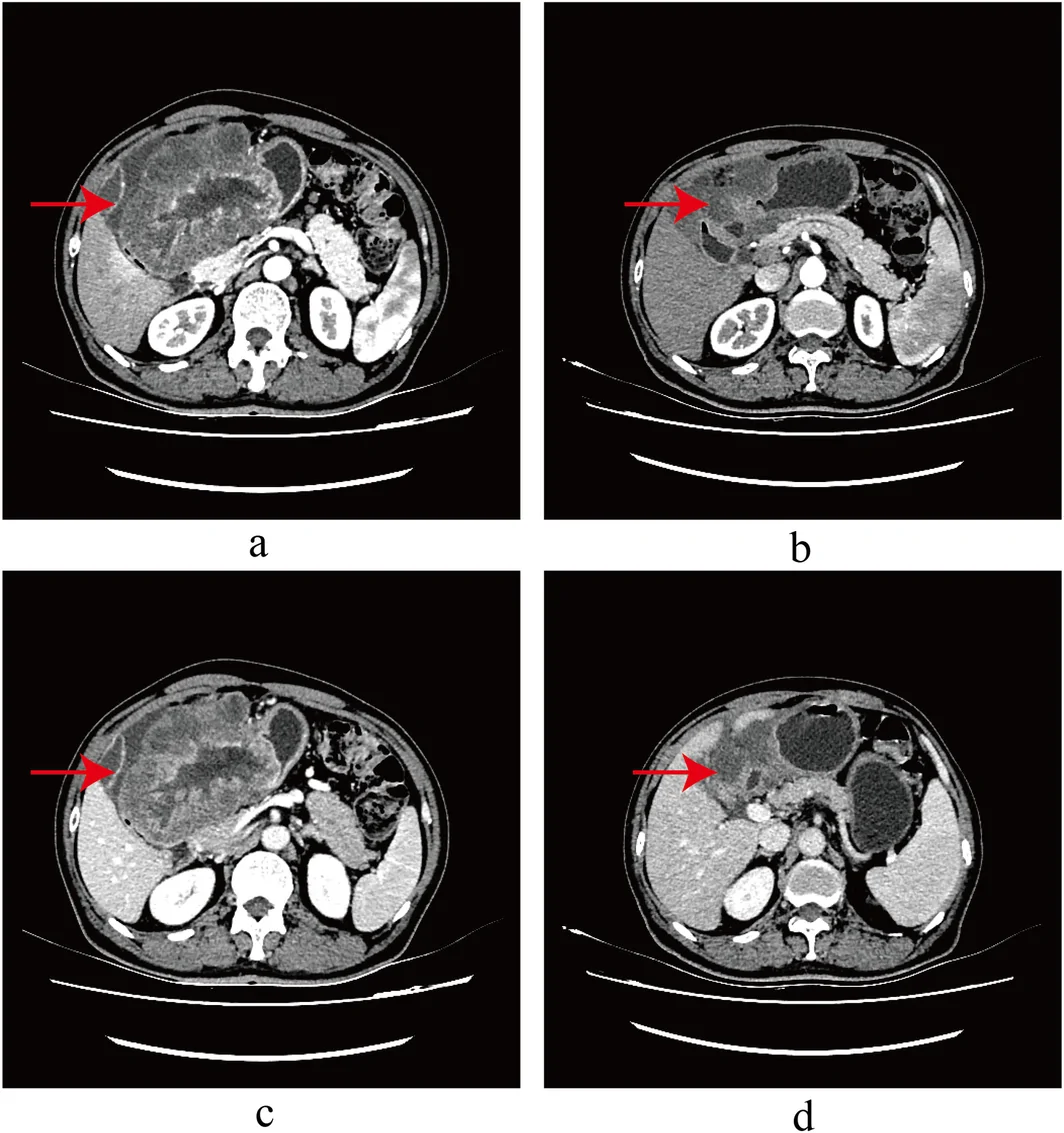
Enhanced abdominal computed tomography images for evaluating the treatment response. a: The arterial phase CT radiomics on July 18, 2023; b: The arterial phase CT radiomics on February 27, 2024; c: The venous phase CT radiomics on July 18, 2023; d: The venous phase CT radiomics on February 27, 2024.

Pretreatment contrast‐enhanced abdominal CT revealed a multilocular cystic‐solid mass, predominantly cystic in nature, measuring approximately 8.9 × 11.3 cm at its largest cross section, with ill‐defined borders between the lesion and the gastric antrum/duodenal wall. The solid components showed heterogeneous enhancement with multiple small feeding vessels, while the cystic components showed no significant enhancement. The right diaphragm appeared focally thickened with a nodular appearance (approximately 1.7 cm), raising concern for possible metastatic involvement. Multiple slightly enlarged lymph nodes were identified in the retroperitoneal and hepatogastric spaces (Figure 3a,c). Gastroscopy showed a deformed antrum with a large, friable ulcerated mass obstructing the lumen; the scope could not pass (Figure 2).

## 3 Differential Diagnosis, Investigations and Treatment

A multidisciplinary team (MDT) comprising general surgery, oncology, radiology, pathology, and pharmacy specialists conducted a comprehensive evaluation. The MDT formally reviewed the pretreatment imaging and concluded that the tumor was unresectable upfront based on the following specific criteria: (1) a right diaphragmatic nodular lesion suspicious for metastasis (M1), which precluded curative intent surgery; (2) retroperitoneal (station 16) lymphadenopathy beyond the standard D2 lymphadenectomy territory; and (3) ill‐defined borders between the primary tumor and the pancreatic head/duodenum, raising concern for adjacent organ invasion that could compromise a negative surgical margin. According to the AJCC 8th edition, the clinical stage was defined as cT4aN2M1 (Stage IV). The patient was young (55 years), ECOG 0, with good organ function, making him a suitable candidate for intensive conversion therapy despite high tumor burden. The MDT specifically noted that the right diaphragmatic lesion and retroperitoneal lymphadenopathy were the key targets for conversion, and that successful downstaging of these lesions would be the prerequisite for subsequent surgical exploration. The MDT recommended Sintilimab + SAP‐SOX (quadruple regimen) to maximize tumor downstaging and permit R0 resection. A detailed toxicity monitoring and supportive care plan was established, and the patient provided informed consent.

Treatment regimen (21‐day cycle): sintilimab 200 mg IV day 1, nab‐paclitaxel 200 mg IV day 1, oxaliplatin 180 mg IV day 1, and oral S‐1 60 mg twice daily for 14 days. Response was assessed by CT after every 2 cycles. After 6 cycles of conversion therapy, contrast‐enhanced CT showed marked regression of the tumor. The overall cystic‐solid mass decreased from 8.9 × 11.3 cm to 4.6 × 6.1 cm in maximum cross section. More importantly, the enhancing solid components, which were defined as target lesions per RECIST 1.1, completely disappeared, with only residual non‐enhancing cystic components remaining. The previously ill‐defined border between the tumor and the pancreatic head/duodenum became clearly delineated. No new lesions or significantly enlarged lymph nodes were observed (Figure 3b,d). Critically, the right diaphragmatic nodule and the retroperitoneal (station 16) lymph nodes showed complete disappearance of enhancing components, indicating loss of metabolic activity in these previously suspicious metastatic lesions. Serum CEA normalized to 4.58 ng/mL. No additional imaging modalities such as PET/CT or diffusion‐weighted MRI were performed; resolution of all enhancing components, resolution of obstructive symptoms and marked clinical response—was considered sufficiently convincing by the MDT to proceed with surgical exploration. Based on these findings—specifically, the loss of enhancement in the right diaphragmatic and retroperitoneal lesions, along with the marked downstaging of the primary tumor—the MDT reassessed the patient and determined that curative‐intent surgery was now feasible. Per RECIST 1.1, the patient achieved clinical complete remission (cCR). With patient consent, laparoscopic distal gastrectomy with D2 lymphadenectomy was performed on February 29, 2024. Intraoperatively, the tumor was located at the pylorus, measuring approximately 6 × 5 cm, with serosal invasion but no grossly enlarged lymph nodes. D2 lymphadenectomy was performed, encompassing stations 5, 6, 7, 8a, 9, 11p, and 12a. A total of 15 lymph nodes were retrieved and examined, all of which were negative for residual disease. Postoperative pathology confirmed R0 resection with negative resection margins (proximal margin 7 cm, distal margin 1.5 cm, both negative for tumor involvement). No residual tumor cells were identified in the gastric wall; only multifocal acellular mucin pools were observed in the submucosa, muscularis propria, and subserosa, with no viable tumor cells in the primary lesion or in any of the 15 retrieved lymph nodes (0/15). The treatment response was graded as chemotherapy response score 0, corresponding to Becker tumor regression grade TRG 1a (complete tumor regression with no viable residual tumor cells), confirming pathological complete remission (pCR, pT0N0M0) (Figure 1b,c). The acellular mucin pools were interpreted according to the Becker system as therapy‐induced regression changes rather than residual disease. This pathological finding also served as a critical validation of the preoperative cCR assessment: the non‐enhancing cystic components observed on posttreatment CT were confirmed to represent therapy‐induced acellular mucin without viable tumor cells, thereby corroborating the clinical response evaluation.

**FIGURE 4:**
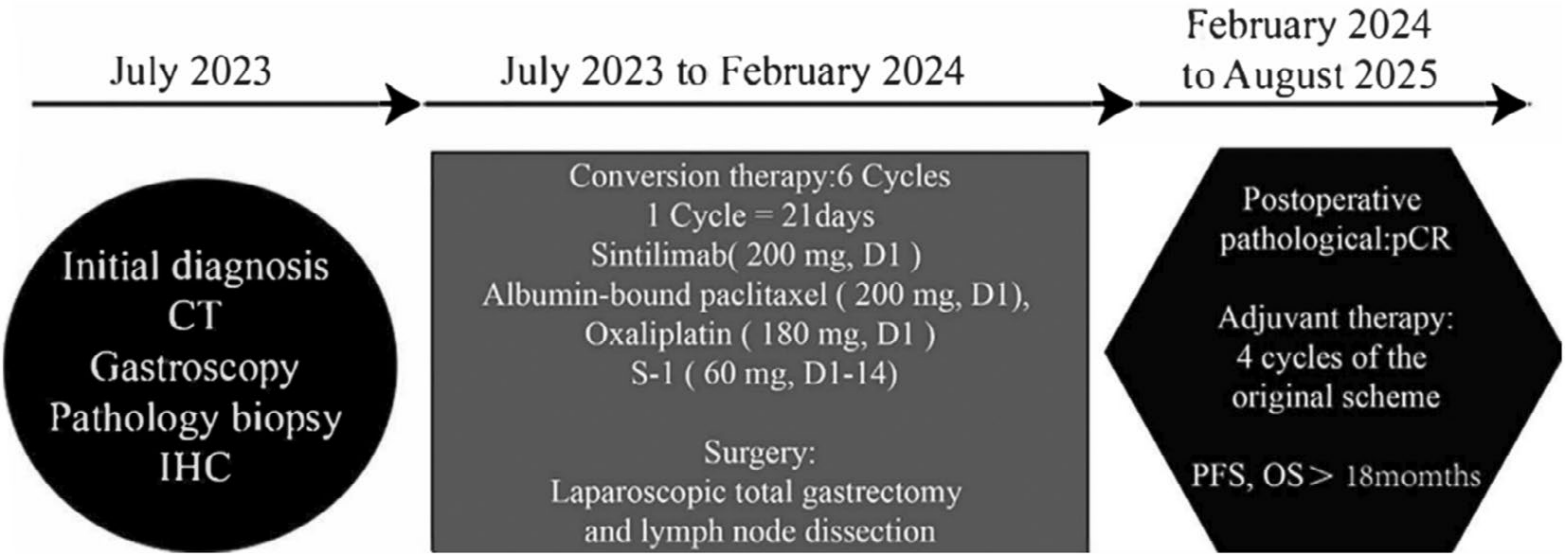
Timeline of treatment options. CT, Computed tomography; IHC, Immunohistochemistry; OS, overall survival; pCR, pathological complete response; PFS, progression‐free survival.

Adjuvant therapy with the same regimen was continued for 4 cycles. At last follow‐up (August 2025), PFS and OS both exceeded 18 months, with good quality of life. During conversion therapy, the patient developed Grade II myelosuppression (neutrophil nadir: 1.2 × 10^9^/L) and Grade I hepatic impairment (ALT peaked at 62 U/L), which were detected on day 1 of cycle 3 and cycle 4, respectively. These were managed with subcutaneous human granulocyte colony‐stimulating factor (160 μg daily) and intravenous diisopropylamine dichloroacetate/sodium gluconate (160 mg daily). No dose modifications or treatment delays were required, as both adverse events resolved within 3 days before the next cycle. The treatment timeline is summarized in Figure 4.

## 4 Conclusion and Results

This case illustrates the potential that sintilimab combined with nab‐paclitaxel and SOX as conversion therapy may achieve deep remission in initially unresectable advanced gastric cancer in a fit patient with favorable tumor immune microenvironment features. The patient experienced rapid CEA normalization, clinical complete response, and ultimately pathological complete remission with R0 resection. As of 18 months post‐diagnosis, the patient remains disease‐free. Owing to inherent limitations of a single case report, these findings are strictly hypothesis‐generating rather than conclusive and should not be interpreted as evidence of superiority of this specific regimen over other established conversion approaches. Larger prospective studies are required to validate the efficacy and safety of this combination and to define optimal patient selection criteria.

## 5 Discussion

Surgical resection remains the only curative option for gastric cancer [2]. In this case, six cycles of Sintilimab + SAP‐SOX conversion therapy enabled R0 resection in a patient with initially unresectable disease. The success stems from precise patient selection and individualized MDT‐guided management. The patient's young age, good performance status (ECOG 0), and absence of major organ dysfunction allowed tolerance of intensive therapy. MDT oversight ensured scientific decision‐making, dynamic toxicity monitoring, and timely intervention (e.g., for myelosuppression), embodying the principle of “right patient, right management” crucial for conversion therapy success [13].

The selection of this specific quadruple regimen was based on several considerations. First, the addition of a PD‐1 inhibitor (sintilimab) to chemotherapy is now a standard first‐line treatment for advanced gastric cancer, as established by Phase III trials including ORIENT‐16 [4]. Second, among available chemotherapeutic backbones for conversion therapy, we chose the SOX regimen (S‐1 plus oxaliplatin) because it is widely used in Asian populations and has demonstrated favorable efficacy and tolerability in the perioperative setting [14]. Third, nab‐paclitaxel was incorporated based on its potential synergistic mechanisms with immunotherapy: beyond its cytotoxic effects through tubulin stabilization, nab‐paclitaxel enhances drug accumulation in tumors via the enhanced permeability and retention effect and may reduce immunosuppressive cells (Tregs, MDSCs) in the tumor microenvironment [15]. Mechanistically, the regimen's efficacy reflects synergistic immunochemotherapy. This triplet combination (PD‐1 inhibitor + SOX + nab‐paclitaxel) was designed to maximize tumor downstaging while maintaining an acceptable safety profile in a fit patient (ECOG 0) with high tumor burden. The rapid CEA decline and pCR achieved align with promising conversion rates reported in CO‐STAR and other studies [11, 12].

Postoperative continuation of the same adjuvant regimen was chosen given the initially high tumor burden, aiming to eradicate minimal residual disease, though its long‐term value requires further investigation. However, it is important to recognize that the observed pCR in this case cannot be attributed solely to this specific drug combination. The patient exhibited multiple favorable characteristics that may independently predict treatment sensitivity and favorable prognosis, including an excellent performance status (ECOG 0), young age (55 years), robust CD8+ T‐cell infiltration (∼20%), a high Ki‐67 proliferation index (∼60%), mucinous histology, and a PD‐L1 CPS of approximately 5. These biological and clinical features may have rendered the tumor intrinsically more susceptible to immune checkpoint blockade and chemotherapy, raising the possibility that alternative PD‐1 inhibitor‐based regimens could have produced comparable results in this biologically favorable context. Therefore, while the Sintilimab + SAP‐SOX regimen represents a reasonable and evidence‐based choice, the favorable outcome should be interpreted primarily as a hypothesis‐generating observation in a highly selected patient, rather than as evidence of superiority of this particular combination. EBV status and tumor mutational burden were not assessed due to institutional practice at the time of diagnosis—an acknowledged limitation of this report.

The decision to administer four additional cycles of the same regimen postoperatively warrants further explanation. Currently, no established consensus supports the routine continuation of the identical intensive immunochemotherapy regimen after achieving pCR and R0 resection in conversion therapy settings. The decision in this case was made through multidisciplinary discussion and was based on several considerations. First, the patient had an exceptionally high baseline tumor burden (CEA > 1100 ng/mL, primary tumor > 8 cm) and initially unresectable Stage IV disease, which raised concerns about potential micrometastatic disease beyond the resected field, despite the favorable pathological response. Second, the patient tolerated the preoperative regimen well, with only Grade I–II toxicities that resolved promptly, making continued treatment clinically feasible. Third, emerging evidence suggests that approximately 6 months of preoperative systemic therapy followed by R0 resection and up to 1 year of postoperative maintenance therapy may be beneficial in conversion therapy settings [16]. However, we acknowledge that this approach remains investigational [17]. The potential risks of prolonged immunochemotherapy—including cumulative neurotoxicity, myelosuppression, hepatic impairment, and immune‐related adverse events—must be weighed against the uncertain benefit of eradicating minimal residual disease. In retrospect, given the achievement of pCR, a de‐escalation strategy (e.g., single‐agent S‐1 or observation) might have been a reasonable alternative. This case highlights the current lack of high‐level evidence to guide postoperative therapy after pCR in conversion therapy, underscoring the urgent need for prospective studies to define optimal adjuvant strategies in this setting.

Several alternative conversion regimens have been investigated. The FLOT regimen combined with a PD‐1 inhibitor (toripalimab) achieved an R0 conversion rate of 25% in a Phase II trial for gastric cancer with peritoneal metastasis [18]. In the CO‐STAR trial, sintilimab combined with nab‐paclitaxel, S‐1, and apatinib reported an R0 conversion rate of 47.2% and a pCR rate of 22.2%, though this regimen included an anti‐angiogenic agent (apatinib), which may increase the risk of bleeding, gastrointestinal perforation, and wound healing complications. By contrast, our Sintilimab + SAP‐SOX approach excludes an anti‐angiogenic agent, potentially offering a safer profile for patients proceeding to major gastric surgery [11]. Another Phase II study of sintilimab plus fruquintinib and SOX showed an R0 conversion rate of 82.9% and a pCR rate of 10.3%, but this regimen also incorporated a multi‐targeted tyrosine kinase inhibitor with similar safety concerns [19]. Additionally, the SOX backbone we selected is widely used and well‐tolerated in Asian populations, making it a practical and established choice for conversion therapy in this demographic [20]. Compared with these regimens, our approach offers a chemotherapy–immunotherapy doublet without an anti‐angiogenic agent, potentially reducing bleeding and wound healing risks while still achieving deep remission. However, direct comparative data are lacking, and the optimal regimen remains to be defined by ongoing prospective trials. Recent studies have explored CT‐based radiomics for distinguishing gastric cancer subtypes and noninvasive PD‐L1 imaging using labeled antibodies, though these techniques remain experimental. In our case, conventional histopathology and IHC‐based PD‐L1 assessment were used to guide treatment decisions [16, 21].

However, the regimen's intensity raises safety concerns. As observed, hematological toxicity and liver impairment are manageable but mandate close monitoring. Thus, this strategy is best reserved for rigorously selected, fit patients [22, 23, 24]. Importantly, while the treatment response in this case was remarkable, the favorable outcome must be interpreted with caution. Several alternative explanations warrant consideration beyond the efficacy of the specific drug combination. First, the patient's tumor exhibited several biological features potentially associated with enhanced immunotherapy sensitivity, including substantial CD8+ T‐cell infiltration (∼20%), a relatively high Ki‐67 proliferation index (∼60%), and mucinous histology. These features may have rendered the tumor intrinsically more susceptible to PD‐1 blockade, independent of the specific chemotherapeutic backbone. Second, the patient's excellent performance status (ECOG 0) and young age (55 years) likely contributed to better tolerance of intensive therapy and more robust immune reconstitution, factors that are often underappreciated in case reports but are critical determinants of outcomes. Third, the baseline PD‐L1 CPS of 5, although not exceptionally high, may still be sufficient to predict responsiveness to sintilimab in the context of chemotherapy‐induced immunogenic cell death, which could amplify the antitumor immune response regardless of the specific regimen used. It is therefore plausible that the observed pCR reflects a combination of favorable tumor biology, host factors, and treatment effects, rather than the superiority of the Sintilimab + SAP‐SOX regimen per se.

The prognostic value of pCR in gastric cancer has been increasingly recognized in the literature. A recent meta‐analysis of 25 studies involving 20,503 patients demonstrated that achieving pCR after neoadjuvant chemotherapy is significantly associated with improved OS (HR = 0.63, 95% CI: 0.43–0.93, *p* = 0.005) and DFS (HR = 0.34, 95% CI: 0.21–0.57, *p* < 0.001) under the Becker tumor regression grading system [25]. Data from the China National Cancer Center showed that patients achieving pCR had 5‐year OS and DFS rates of 83.3% and 82.0%, respectively [26]. These findings suggest that pCR is not merely a histological endpoint but a strong surrogate for long‐term survival. In our patient, the achievement of Becker TRG 1a (chemotherapy response score 0) therefore carries favorable prognostic implications, although the relatively short follow‐up of 18 months precludes definitive conclusions regarding cure.

Single‐case observations are inherently susceptible to selection bias and cannot establish a causal relationship between a specific drug combination and treatment outcomes. Consequently, our report serves primarily to generate hypotheses for future biomarker‐driven studies rather than to provide practice‐changing evidence. Currently, evidence for gastric cancer conversion therapy derives mainly from Phase II or retrospective studies; high‐level data are lacking [24, 27]. We acknowledge several limitations. The follow‐up period of 18 months, though encouraging, remains insufficient to establish long‐term cure. The absence of comprehensive molecular profiling (e.g., EBV status, TMB) limits the generalizability of our findings. Additionally, the favorable baseline characteristics of this patient may not reflect the broader population with unresectable gastric cancer. Ongoing prospective trials (e.g., NCT04889768, NCT04510064, NCT05648487) will help identify optimal candidates, treatment duration, and toxicity management pathways [28, 29, 30].

In summary, this case suggests that for carefully selected patients with initially unresectable advanced gastric cancer, sintilimab combined with nab‐paclitaxel and SOX as conversion therapy can yield deep remission and radical resection. It also highlights the critical role of MDT in balancing efficacy and safety. Future research will refine precision and standardization of this approach.

## Author Contributions

**Yufan Tang:** conceptualization, investigation, visualization, writing – original draft, writing – review and editing. **Boquan Zhou:** conceptualization, investigation, writing – review and editing. **Bingbing Wen:** investigation, writing – review and editing. **Ke Yu:** investigation, visualization, writing – review and editing. **Jiajia Jia:** investigation, visualization. **Ying Sha:** investigation, visualization. **Jixiang Liu:** investigation, visualization. **Luyao Li:** investigation, visualization. **Ruifang Fan:** conceptualization, funding acquisition, project administration, supervision, writing – review and editing.

## Funding

The study was supported by the Natural Science Foundation of Gansu Province (22JR5RA004) and the Scientific Research Plan Project of the 940th Hospital of Joint Logistics Support Force (2021yxky045).

## Consent

Written informed consent was obtained from the patient for publication of this article and any accompanying images.

## Conflicts of Interest

The authors declare no conflicts of interest.

## Data Availability

The relevant information of this case can be obtained by the corresponding author under reasonable requirements.
